# ﻿New records of leaf-miner flies (Diptera, Agromyzidae) from Morocco

**DOI:** 10.3897/zookeys.1230.141900

**Published:** 2025-03-05

**Authors:** Mourad Doukale Daief, Michael von Tschirnhaus, Kawtar Kettani

**Affiliations:** 1 Laboratory of Ecology, Systematics and Conservation of Biodiversity (LESCB), URL-CNRST N°18, FS, Abdelmalek Essaadi University, Tetouan, Morocco Abdelmalek Essaadi University Tetouan Morocco; 2 Faculty of Biology, Biological Collection, University of Bielefeld, P.O. Box 100 131, 33501 Bielefeld, Germany University of Bielefeld Bielefeld Germany

**Keywords:** Agromyzidae, faunistics, flies, Moroccan fauna

## Abstract

New records are provided of the family Agromyzidae (leaf-miner flies) from Morocco. Thirty agromyzid species are newly recorded, seven of these in Agromyzinae: *Agromyza* (*Agromyzaanthracina*, *A.conjuncta*, *A.mobilis*, *A.myosotidis*, *A.nigrescens*), *Hexomyza* (*H.simples*), and *Ophiomyia* (*O.pinguis*). The other 23 new records are in Phytomyzinae: Amauromyza (Cephalomyza) karli, *Calycomyzaflavomaculata*, *C.solidaginis*, Cerodontha (Butomomyza) eucaricis, C. (Dizygomyza) luctuosa, C. (Icteromyza) geniculata, *Chromatomyiacentaurii*, *Liriomyzaamoena*, *L.brassicae*, *L.strigata*, *Phytoliriomyzaarctica*, *P.perpusilla*, *P.pteridii*, *Phytomyzaanemones*, *Ph.clematidis*, *Ph.crassiseta*, *Ph.notata*, *Ph.plantaginis*, *Ph.rufipes*, *Ph.vitalbae*, *Pseudonapomyzaconfusa*, *Ps.palavae*, and *Ps.vota*. The genus *Calycomyza* Hendel is newly reported from the country. The present study is based on recent entomological surveys and has increased the total number of agromyzid species in Morocco to 92. Information on the distribution and ecology of each species is provided.

## ﻿Introduction

The family Agromyzidae, or leaf-miner flies (Diptera, Acalyptratae), is widely distributed in the Palaearctic Region, in which it comprises more than 1,303 species (von Tschirnhaus and Groll 2024a, 2024b). Of these, 984 species are recorded from Europe (Černý, pers. comm. to MvT, 24.ix.2024). They are small to medium-sized acalyptratae flies (0.8–6.5 mm), with variable colour, from black to yellow. They are considered as the third largest of Acalyptratae families which possess entirely phytophagous larvae that develop in a wide range of hosts in the plant kingdom (Hering 1957, 1966; Spencer 1972, 1990). They inhabit a wide variety of habitats in forests, high mountains, arctic tundra and all types of lowlands, wetlands, and marine coasts. They play a major role in agricultural entomology and are therefore of economic interest (Spencer 1973), as larvae of many species are known to damage crop plants (Dempewolf 2004; Lonsdale et al. 2023). This family of Diptera is also of significant ecological importance (Spencer 1973). The larvae of most species are monophagous or oligophagous, but polyphagy is the exception. They attack different parts of plants such as leaves, stems, sometimes the roots, flower heads, seeds and the young xylem of shrubs and trees, erroneously addressed by authors as the cambium. After host plant identification, the form of the leaf-mine can be useful to identify the species that caused the damage (Černý et al. 2018).

In Morocco, the first record of the family Agromyzidae was reported by Becker and Stein (1913) who found *Amauromyzamorionella* [at that time included in the genus *Agromyza*] at Tangier. After the 1930s, new records were added by Séguy’s study of the Diptera of Morocco. He reported seven species for the country (Séguy 1936, 1953) with a few other records by Hendel (1931–1936) and Kozlowsky and Rungs (1932). Only three species have been described from Morocco after 1936. These are *Aulagromyzacydoniae* [described by Hendel (1936) in combination with *Phytagromyza*], *Aulagromyzaatlantidis* [described by Spencer (1967) in combination with *Paraphytomyza*], and Cerodontha (Icteromyza) rozkosnyi described by Černý (2007). It was not until the 1960s that more records were made with Spencer’s (1967, 1973) studies on the Agromyzidae of Morocco and whose contribution had slightly improved the knowledge of these flies by adding 17 species to the Moroccan fauna. After that, few further records have been added by foreign researchers such as Griffiths (1974, 1967), Geipert et al. (1994), Maarouf (2003), and Hanafi and Schnitzler (2004). The most important contribution to the improvement of our knowledge of Moroccan Agromyzidae was made recently by Černý through his many studies (Černý 2007, 2009, 2010, 2012, 2013, 2019; Černý and Merz 2006, 2007; Černý and von Tschirnhaus 2014; Černý et al. 2020). Those articles provided a further 20 species to the country. Beyond that, our knowledge of Moroccan agromyzid fauna is still poor and many gaps remain for this diverse family of Diptera. Many regions of Morocco were still unexplored such as the Rif region in northern Morocco. Indeed, only 62 species of Agromyzidae are listed so far in the catalogue of the Diptera of Morocco compiled by Kettani et al. (2022). Of these, only seven species were recorded from the Rif.

The aim of this study is to present new faunistic records, in particular from some regions which have not previously been explored as an attempt to fill gaps in our knowledge of Moroccan leaf-miners.

The distribution of the species given in this study was mainly gathered from the literature provided by Papp and Černý in their four volumes (Papp and Černý 2015, 2016, 2017, 2020 [as 2019]), Černý et al. 2020; Lonsdale 2021, Lonsdale et al. 2023, and using the file cards and bibliography for world Agromyzidae in von Tschirnhaus and Groll (2024a, 2024b).

## ﻿Materials and methods

**Figure 1.**
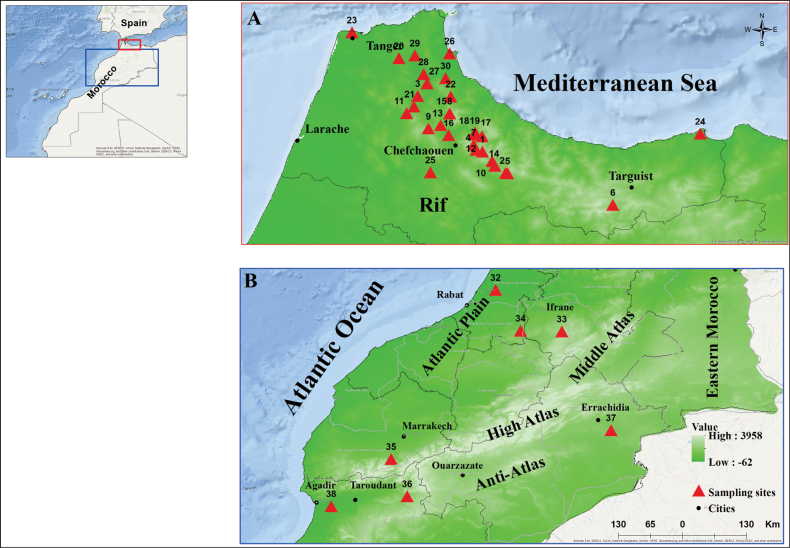
Location of the studied sites in Morocco **A** the Rif at the north of Morocco **B** central Morocco with Atlantic Plain and Atlas Mountains.

The specimens examined in the present study were collected by MD and to a large extent by KK and her students. Insects were captured by sweep net and by Malaise trap at different localities throughout Morocco from 2012 to 2023. Of the 140 sites surveyed, 38 were home to the agromyzid species mentioned in this article. The sites are mainly in the Rif and Atlas Mountains region (Fig. 1) where a variety of habitats including forests, grasslands, riverbanks, and wetlands were prospected. Table 1 summarizes the main geographical cartographic data and types of habitats of the prospected sites.

**Table 1.** Localities with respective biogeographical areas, coordinates and altitudes of the studied sites.

Code of locality	Biogeographic area, locality, collecting site (protected area)	Geographic coordinates	Altitude (m)	Habitat
1	Rif, Talassemtane, Maison forestière (NPTL)	35°8'6.11"N, 5°8'18.06"W	1696	Fir forest
2	Rif, Chefchaouen, Bab Tariouant	35°01'7.68"N, 5°0'35.999"W	1429	Oak forest (*Quercuspyrenaica*)
3	Rif, Tarzout, Riba (PNPB)	35°15'755"N, 5°99'691"W	1421	Mixed forest
4	Rif, Talassemtane, Place Espagna (NPTL)	35°05'938"N, 5°08'956"W	1386	Fir forest
5	Rif, El Anasser, Dayat El Anasser	35°00'59.8"N, 4°59'59.77"W	1383	Around pond
6	Rif, Targuist, Beni Bounsar	34°50'36"N, 4°25'23"W	1340	Cedar forest
7	Rif, Afeska, Oued Afeska (NPTL)	35°10'18.4"N, 5°11'10.5"W	1293	Banks of mountain stream
8	Rif, Jbel Kelti, Oued Tanina	35°20'45.50"N, 5°19'01.70"W	1195	Banks of mountain stream
9	Rif, Bouhachem, Amsemlil (PNPB)	35°15'44.424"N, 5°26'0.276"W	1059	Edges of mountain peat bog
10	Rif, Ametrasse, Oued Ametrasse	35°05'01"N, 5°05'03"W	841	Riverbank
11	Rif, Tazia, Dayat Tazia	35°20'52"N, 5°33'12"W	720	Edges of pond
12	Rif, Akchour, Chellal Kbir (NPTL)	35°13'8"N, 5°8'19"W	680	Banks of waterfall
13	Rif, Bni Hassan, Triwa	35°16'50.7"N, 5°22'01.6"W	654	Meadow
14	Rif, Bni Darkoul, Oued Souk Lhad	35°3'26"N, 5°4'10"W	613	Riverbank
15	Rif, Chefchaouen, Bni Ahmed	35°20'45.50"N, 5°19'01.70"W	590	Agricultural field
16	Rif, Tazrout, Adrou (PNPB)	35°13'32.30"N, 5°19'24.35"W	556	Mixed forest
17	Rif, Akchour, Pont de Dieu (NPTL)	35°13'42"N, 5°10'31"W	536	Riverbank
18	Rif, Akchour, Chellal Sghir (NPTL)	35°14'21"N, 5°10'37"W	424	Banks of waterfall
19	Rif, Talembote, Oued Talembote (NPTL)	35°14'20.586"N, 5°10'42.037"W	405	Riverbank
20	Rif, Fahs-Anjra, Rmel	35°38'57.4"N, 5°35'41.4"W	372	Riverbank
21	Rif, El Hamma, Masjid El Hamma	35°23'6"N, 5°30'46"W	330	Matorral dominated by cork oak trees
22	Rif, Tétouan, Oued Raouz	35°26'23.03"N, 5°18'41.70"W	265	Matorral dominated by pine trees
23	Rif, Rmilat, Parc Perdicaris	35°47'26.675"N, 5°51'12.938"W	223	Urban park
24	Rif, Oulad Sidi Mansour, Oued Tassikeste	35°14'05"N, 3°56'37"W	144	Riverbank
25	Rif, Souk El Had, Oued El Koub	35°1'17.88"N, 5°25'19.98"W	124	Riverbank
26	Rif, M’Diq, Koudiat Taifour	35°40'28.706"N, 5°19'1.841"W	100	Pine forest
27	Rif, Ben Karrich, Oued Mhajrate	35°30'39"N, 5°26'27"W	67	Riverbank
28	Rif, Tétouan, Khemis Anjra	35°33'42.453"N, 5°27'34.246"W	63	Riverbank
29	Rif, Tétouan, Oued Khemis	35°39'51.00"N, 5°30'29.00"W	61	Riverbank
30	Rif, Kitane, Oued Zarka	35°32'24.72"N, 5°20'23.58"W	49	Riverbank
31	Eastern Morocco, Taourirt, El Aïoun Sidi Mellouk	34°35'25.336"N, 2°31'4.263"W	589	Agricultural field
32	Atlantic Plain, Sidi Yahya Gharb, forest of Sidi Yahya Gharb	34°18'16"N, 6°18'38"W	20	Eucalyptus forest
33	Middle Atlas, Ifrane, Lac Zarouka (NPIF)	33°32'36.163"N, 5°5'45.277"W	1615	Edges of mountain lake
34	Middle Atlas, Ifrane, Zaouiet Ifrane (NPIF)	33°33'32.884"N, 5°51'0.31"W	1535	Cedar forest
35	High Atlas, Al Haouz, Amizmiz (NPTB)	31°12'54.93"N, 8°13'6.14"W	991	Mixed forest
36	Anti-Atlas, Taroudant, Taliouine	30°31'56"N, 7°55'27"W	928	Agricultural field
37	Anti-Atlas, Errachidia, Aoufous	31°44'31.607"N, 4°11'54.896"W	906	Agricultural field
38	Anti-Atlas, Agadir Idaoutanane, Vallée du Paradis	30°21'6.84"N, 9°18'46.8"W	587	Riverbank

The Rif region consists of a mountainous domain overlooking the Mediterranean Sea at the north of Morocco. The climate is mainly of the Mediterranean type and is characterized by high levels of precipitation and is considered very rich and diverse in vegetation cover (Benabid 1982; Valdés Castrillón et al. 2002). It integrates five bioclimatic stages: per-humid and humid which are mainly restricted to the high mountain summits, while the subhumid, semi-arid and arid parts are more widespread in the rest of the Rif. The Atlas region consists of mountain massifs located in the centre and further south of Morocco, made up of three large mountain ranges: High Atlas, Middle Atlas and Anti-Atlas with high summits incised by deep valleys. The bioclimates of this area include perhumid, humid, sub-humid, semi-arid and arid zones with more than 800 mm of annual precipitation on the highest peaks (HCEFLCD 2017) which have favoured a great diversity of vegetation such as *Tetraclinisarticulata*, *Arganiaspinosa*, *Quercussuber*, *Quercusfaginea*, *Quercusilex*, *Cedrusatlantica*, and *Juniperusthurifera* (Mokhtari et al. 2014).

Most specimens captured in the field were stored in 70% ethanol until identification. Each specimen was examined in detail before and after its genitalia were mounted. The genitalia, cut off from the rest of the abdomen, were cleared in 10% KOH at 80 °C for 8 or 10 min and then transferred into 10% acetic acid for ~ 5 min, and finally placed in a droplet of glycerine. The genitalia are firmly embedded in glycerine on glass-slides which were sealed by resistant nail varnish to protect them from dust and from drying out. All species have been identified based on the male genitalia. The second author confirmed and validated all the species identifications by checking all photographs.

Specimens are housed in the Laboratory of Ecology, Systematics and Conservation of the Biodiversity (**LESCB**) in the Faculty of Sciences of Tetouan in the personal collection of Mourad DOUKALE DAIEF.

The following abbreviations are used for the material examined:

**NPIF** National Park of Ifrane

**NPTB** National Park of Toubkal

**NPTL** National Park of Talassemtane

**PNPB** Project of Natural Park of Bouhachem

## ﻿Results

### ﻿Subfamily Agromyzinae

#### ﻿ Agromyza anthracina

Taxon classificationAnimaliaDipteraAgromyzidae

Meigen, 1830

728ACB6C-EA03-51A1-AC69-185B50FE9EA5

##### Material examined.

Morocco. • 1 ♂; Rif, M’Diq, Koudiat Taifour; 3 Apr. 2018; K. Kettani leg.; sweep net; LESCB-R18/21.

##### Distribution.

Palaearctic species, widespread in Europe. First record from Morocco.

##### Habitat.

Collected in pine forest (*Pinuspinaster*).

#### ﻿ Agromyza conjuncta

Taxon classificationAnimaliaDipteraAgromyzidae

Spencer, 1966

DBF03840-59F3-58B5-9325-D6A255F5A862

##### Material examined.

Morocco. • 2 ♂♂; Rif, Souk El Had, Oued El Koub; 23 Apr. 2018; K. Kettani leg.; sweep net; LESCB-R18/23. • 1 ♂; Rif, Bni Hassan, Triwa; 8 May 2018; K. Kettani leg.; sweep net; LESCB-R18/25. • 1 ♂; Rif, Tazrout, Riba (PNPB); 29 Apr.–22 May 2019; K. Kettani leg.; Malaise trap; LESCB-R19/28.

##### Distribution.

Great Britain, Greece (Crete), Hungary, Romania, Serbia, Italy (Sicily), Slovakia and Spain. First record from Morocco.

##### Habitat.

This species has a large distribution in the Rif Region across a wide altitudinal range (124–1421 m). It was found on the banks of wetlands bordered by *Ericaarborea*, *Pistacialentiscus*, and *Rubusulmifolius*, in a meadow dotted by Asteraceae and Poaceae and in a mixed oak forest (*Quercussuber*, *Quercuscanariensis*).

#### ﻿ Agromyza mobilis

Taxon classificationAnimaliaDipteraAgromyzidae

Meigen, 1830

69CBF644-93C9-5C6E-A00B-E56452B9CD60

##### Material examined.

Morocco. • 1 ♂; Rif, Akchour, Chellal Sghir (NPTL); 7 June–14 July 2017; K. Kettani leg.; Malaise trap; LESCB-R19/29.

##### Distribution.

Andorra, Belgium, Czech Republic, Denmark, Estonia, Finland, France (including Corsica), Germany, Great Britain, Greece (Dodecanese, Rhodes), Hungary, Italy, Japan, Latvia, Lithuania, Netherlands, Norway, Poland, Portugal, Russia, Slovakia, Spain, Sweden, Switzerland, Turkey, Ukraine, and Yugoslavia. First record from Morocco.

##### Habitat.

Banks of waterfall lined by *Pistacialentiscus*, *Neriumoleander*, *Ficuscarica*, *Mentha* sp., and *Thuya* sp.

#### ﻿ Agromyza myosotidis

Taxon classificationAnimaliaDipteraAgromyzidae

Kaltenbach, 1864

E63940B4-5927-5FDE-BEC7-5052A8F1A57E

##### Material examined.

Morocco. • 1 ♂; Rif, El Hamma, Masjid El Hamma; 6–21 June 2016; K. Kettani leg.; Malaise trap; LESCB-R16/10.

##### Distribution.

Belgium, Bulgaria, Czech Republic, Denmark, Finland, Germany, Great Britain, Hungary, Kashmir, Malta, Poland, Spain incl. Canary Islands, Sweden, Switzerland, Turkey and Ukraine. First record from Morocco.

##### Habitat.

Collected in maquis composed of *Quercuscoccifera*, *Oleaeuropaea*, *Fraxinusangustifolia*, *Phillyreaangustifolia*, *Dittrichiaviscosa*, *Pistacialentiscus*, and *Ericaarborea*.

#### ﻿ Agromyza nigrescens

Taxon classificationAnimaliaDipteraAgromyzidae

Hendel, 1920

47340200-F9C0-58F6-A179-3ABCFD464F9F

##### Material examined.

Morocco. • 1 ♂; Rif, Rmilat, Parc Perdicaris; 16 May 2015; K. Kettani leg.; sweep net; LESCB-R15/06.

##### Distribution.

Austria, Belgium, Croatia, Czech Republic, Denmark, Estonia, Finland, Germany, Great Britain, Hungary, Iraq, Italy, Japan, Kyrgyzstan, Lithuania, Malta, Norway, Poland, Romania, Russia, Serbia, Slovenia, Spain incl. Canary Islands, Sweden, Switzerland, and Turkey. First record from Morocco.

##### Habitat.

This species was collected in the forest of Perdicaris Park, at an altitude of 223 m. The habitat is a preserved forest with significant natural and replanted vegetation such as *Oleaeuropaea*, *Cupressusmacrocarpa*, *Quercuscanariensis*, *Pittosporumundulatum*, *Quercussuber*, *Quercuslusitanica*, and *Acacialongifolia*.

#### ﻿ Hexomyza simplex

Taxon classificationAnimaliaDipteraAgromyzidae

(Loew, 1869)

08830B44-CE46-5F0D-AF10-B0BBEA40314E

##### Material examined.

Morocco. • 1 ♂; Anti-Atlas, Errachidia, Aoufous; 26 Sept. 2022; S. Fekrani leg.; sweep net; LESCB-AA22/42.

##### Distribution.

This Holarctic species is known from Albania, Austria, Great Britain, Denmark, Finland, France, Germany, Greece, Hungary, Israel, Italy, Netherlands, Poland, Turkey, Ukraine and in the USA from California, Connecticut, Hawaii, Iowa, Massachusetts, Michigan, and New Jersey. First record from Morocco.

##### Habitat.

This species was found at medium altitude in agricultural fields in arid areas in the south of Morocco.

#### ﻿ Ophiomyia pinguis

Taxon classificationAnimaliaDipteraAgromyzidae

(Fallén, 1820)

695A7AB1-EF47-507E-B78C-B12DA029DC8A

##### Material examined.

Morocco. • 8 ♂♂, 7 ♀♀; Rif, Tazrout, Adrou (PNPB); 14 July–15 Aug. 2013; K. Kettani leg.; Malaise trap; LESCB-R13/02. • 1 ♂; Rif, Kitane, Oued Zarka; 2 Dec. 2017; K. Kettani leg.; sweep net; LESCB-R17/19.

##### Distribution.

Recorded from Austria, Belgium, Great Britain, Bulgaria, China, Croatia, Czech Republic, Denmark, Egypt, Finland, France, Germany, Greece, Hungary, Israel, Italy, Kashmir, Kyrgyzstan, Lithuania, Netherlands, Norway, Poland, Romania, Russia, Slovakia, Spain, Sweden, Switzerland, Tajikistan, Turkey, Ukraine, Uzbekistan, and Yugoslavia. First record from Morocco.

##### Habitat.

This species was collected in a mixed forest (*Quercussuber*, *Pinuspinaster*) and on the banks of a lowland stream (Oued Zarka) bordered by *Neriumoleander*.

### ﻿Subfamily Phytomyzinae

#### ﻿ Amauromyza (Cephalomyza) karli

Taxon classificationAnimaliaDipteraAgromyzidae

(Hendel, 1927)

47FF48D0-F2A7-5159-80C0-31F965AE2309

##### Material examined.

Morocco. • 1 ♂, Rif, Kitane Oued Zarka; 2 Dec. 2017; K. Kettani leg.; sweep net; LESCB-R17/20.

##### Distribution.

Recorded from **Canada**: AB, BC*, MB, NS*, ON, QC, SK*. **USA**: MD*. Europe: Austria, Croatia, Czech Republic, Great Britain, Finland, France, Germany, Greece, Hungary, Korea, Netherlands, Poland, Romania, Russia, Serbia, Slovakia, Spain, Sweden, Switzerland, and Ukraine. China. Mongolia, South Korea. First record from Morocco.

##### Habitat.

This species was collected in one site at low altitude (49 m) on the banks of a lowland stream (Oued Zarka) bordered by *Neriumoleander*.

#### ﻿ Calycomyza flavomaculata

Taxon classificationAnimaliaDipteraAgromyzidae

(Spencer, 1960)

466D8A56-2D97-50C8-BF18-107A15A615E2

##### Material examined.

Morocco. • 1 ♂; Rif, Akchour, Chellal Kbir (NPTL); 25 Apr.–6 June 2016; K. Kettani leg.; Malaise trap; LESCB-R16/12.

##### Distribution.

Croatia, Greece, and Spain. First record from Morocco.

##### Habitat.

Collected on the banks of the Akchour waterfall, lined by *Pistacialentiscus*, *Neriumoleander*, *Ficuscarica*, *Mentha* sp., and *Thuya* sp.

#### ﻿ Calycomyza solidaginis

Taxon classificationAnimaliaDipteraAgromyzidae

(Kaltenbach, 1869)

8D4C1E35-C052-55DD-AEEC-576CFC374A86

##### Material examined.

Morocco. • 1 ♂; Rif, Jbel Kelti, Oued Tanina; 6 June 2021; L. Zouhair leg.; sweep net; LESCB-R21/38.

##### Distribution.

China, Germany, Hungary, Lithuania, Poland, Sweden, Switzerland, Yemen, and **Canada**: AB, NB, NS, ON, QC, YT. **USA**: widespread outside of AK and HI. First record from Morocco.

##### Habitat.

Collected on the banks of a mountain river bordered by *Pistacialentiscus*, *Juniperusoxycedrus*, *Tetraclinisarticulata* and crossing a mixed forest dominated by *Quercussuber*, *Pinuspinaster*, and *Pinushalepensis*. A wide variety of shrubs and herbaceous plant species (*Prunuslusitanica*, *Stachysfontqueri*, *Paeoniacoriacea*, *Eryngiumcaespitiferum*, *Eryngiumtriquetrum*, *Digitalislaciniata*, *Ptilostemonrhiphaeus*, *Ruscushypophyllum*, *Astragalusarmatus*, *Merenderafilifolia*, *Viola* sp.) occupy the undergrowth.

#### ﻿ Cerodontha (Butomomyza) eucaricis

Taxon classificationAnimaliaDipteraAgromyzidae

Nowakowski, 1967

DE4D6EEA-EF37-5487-AA86-787D5A267280

##### Material examined.

Morocco. • 1 ♂; Anti-Atlas, Taroudant, Taliouine; 2 Dec. 2017; Y. Fekrani leg.; sweep net; LESCB-AA17/14.

##### Distribution.

It is known from Belgium, Great Britain, Canada, Czech Republic, Denmark, Finland, France, Germany, Greece, Hungary, Japan, Lithuania, Poland, Slovakia, Sweden, and Switzerland. First record from Morocco.

##### Habitat.

It was collected in an agricultural field bordered by *Carexacuta* and *Carexnigra* in an arid area.

#### ﻿ Cerodontha (Dizygomyza) luctuosa

Taxon classificationAnimaliaDipteraAgromyzidae

(Meigen, 1830)

7BD00638-6BE5-5AF9-9730-26C62CD7C591

##### Material examined.

Morocco. • 1 ♂, 1 ♀; Rif, El Anasser, Dayat El Anasser; 22 Mar. 2019; K. Kettani leg.; sweep net; LESCB-R19/30.

##### Distribution.

Species known from Alaska, Albania, Austria, Belarus, Belgium, Canada, Great Britain, Bulgaria, Byelorussia, Czech Republic, Denmark, Finland, France, Germany, Greece, Hungary, Iraq, Ireland, Israel, Italy including Sicily, Latvia, Lithuania, Netherlands, Norway, Poland, Portugal, Romania, Russia, Slovakia, Spain, Sweden, Switzerland, Tunisia, Uzbekistan, Yugoslavia, and USA (Oklahoma). First record from Morocco.

##### Habitat.

This species was collected in high altitude on the edge of a pond bordered by *Quercuspyrenaica*.

#### ﻿ Cerodontha (Icteromyza) geniculata

Taxon classificationAnimaliaDipteraAgromyzidae

(Fallén, 1823)

0EE7FD15-7A14-5FFF-95DC-AC5FFB6B7324

##### Material examined.

Morocco. • 1 ♂; Middle Atlas, Ifrane, Lac Zarouka (NPIF); 13 Oct. 2023; M. Doukale leg.; sweep net; LESCB-MA23/46.

##### Distribution.

A widespread species. It has been recorded from Albania, Austria, Belarus, Great Britain, Bulgaria, Czech Republic, Denmark, Estonia, Finland, France, Germany, Greece, Hungary, Italy, Latvia, Lithuania, and Macedonia. First record from Morocco.

##### Habitat.

This species was collected on the edge of a mountain lake lined by *Eriophorumlatifolium*, *Populusalba*, *Carex* sp., *Juncusbufonius*, *Schoenoplectuslacustris*, *Typhalatifolia*, and *Salix* sp.

#### ﻿ Chromatomyia centaurii

Taxon classificationAnimaliaDipteraAgromyzidae

Spencer, 1990

86280ECC-8BAB-533D-8383-FD3E235B4E0E

##### Material examined.

Morocco. • 1 ♂; Rif, Akchour, Pont de Dieu (NPTL); 1 July 2019; M. Nourti leg.; sweep net; LESCB-R19/32.

##### Distribution.

Known from Great Britain, Germany, Greece, Hungary, Ireland, Lithuania, and Poland. First record from Morocco.

##### Habitat.

This species was found near running water (Akchour) lined by *Neriumoleander*, *Erica* sp., and *Cistus* sp.

#### ﻿ Liriomyza amoena

Taxon classificationAnimaliaDipteraAgromyzidae

(Meigen, 1830)

0E19885C-8F34-5203-8C25-B024F4993B9C

##### Material examined.

Morocco. • 3 ♂♂, 2 ♀♀; Rif, Khemis Anjra, Oued Khemis; 16 Sep. 2012; K. Kettani leg.; sweep net; LESCB-R12/01.

##### Distribution.

The species is recorded from Austria, Belgium, Great Britain, Bulgaria, Czech Republic, Denmark, Finland, France, Germany, Greece, Ireland, Italy, Japan, Latvia, Lithuania, Moldavia, Netherlands, Poland, Portugal (Madeira Island), Romania, Russia, Serbia, Slovakia, Slovenia, Spain, Sweden, Turkey, and in the Oriental Region (India). First record from Morocco.

##### Habitat.

Collected on the bank of a lowland river bordered by *Neriumoleander*, *Phragmitesaustralis*, *Tamarixafricana*, *Rubusulmifolius*, and *Juncusacutus*.

#### ﻿ Liriomyza brassicae

Taxon classificationAnimaliaDipteraAgromyzidae

(Riley, 1884)

92BBF7A3-A0A8-509D-8CFD-898D93310688

##### Material examined.

Morocco. • 1 ♂; Rif, Souk El Had, Oued El Koub; 23 Apr. 2018; K. Kettani leg.; sweep net; LESCB-R18/24.

##### Distribution.

Wide distribution as an agricultural pest, including in the Afrotropical Region: Cape Verde Islands, Ethiopia, Kenya, Mozambique, Namibia, Oman, Senegal, Spain (Canary Islands), South Africa, Yemen, and Zimbabwe. In the Palaearctic Region including Egypt, France (Corsica), Germany, Iraq, Japan, Malta, Poland, Portugal, Romania, Saudi Arabia, Spain, and Turkey. First record from Morocco.

##### Habitat.

The male was captured on the banks of a river bordered by *Ericaarborea*, *Pistacialentiscus* and *Rubusulmifolius*.

#### ﻿ Liriomyza strigata

Taxon classificationAnimaliaDipteraAgromyzidae

(Meigen, 1830)

BD5D05E4-9A35-5E10-B10E-96DCD4BABD1C

##### Material examined.

Morocco. • 2 ♂♂; Anti-Atlas, Agadir Idaoutanane, Vallée du Paradis; 4 Dec. 2017; Y. Fekrani leg.; sweep net; LESCB-AA17/15.

##### Distribution.

Ubiquitous polyphagous species. It is recorded from Albania, Belarus, Belgium, Bosnia-Herzegovina, Great Britain, Czech Republic, Denmark, Estonia, Finland, France, Germany, Greece, Hungary, Iraq, Ireland, Italy, Kazakhstan, Kyrgyz Republic, Lithuania, Netherlands, Portugal (Madeira Isl.), Norway, Poland, Romania, Russia, Slovakia, Spain, Sweden, Switzerland, Turkey, Ukraine, and Uzbekistan. First record from Morocco.

##### Habitat.

This species was collected on the sandy riverbank in an arid region.

#### ﻿ Phytoliriomyza arctica

Taxon classificationAnimaliaDipteraAgromyzidae

(Lundbeck, 1901)

EA97FC4D-9FD5-58AA-B381-B090905DBD3B

##### Material examined.

Morocco. • 1 ♂; Rif, Chefchaouen, Bni Ahmed; 10 May 2021; K. Kettani leg.; sweep net; LESCB-R21/39.

##### Distribution.

Known from Brazil, Chile, Greenland, North America. Europe. Iran, South Korea, Taiwan, and Sri Lanka. First record from Morocco.

##### Habitat.

It was collected in a *Cannabis* cultivation field with *Cannabissativa* and *Cannabisindica*.

#### ﻿ Phytoliriomyza perpusilla

Taxon classificationAnimaliaDipteraAgromyzidae

(Meigen, 1830)

308E9530-B093-5081-911A-CF0F8B013793

##### Material examined.

Morocco. • 1 ♂; High Atlas, Al Haouz, Amizmiz (NPTB); 26 Mar. 2017; Y. Fekrani leg.; sweep net; LESCB-HA17/16.

##### Distribution.

Known from Albania, Austria, Great Britain, Bulgaria, Cape Verde Islands, Czech Republic, Estonia, Finland, France, Germany, Greece, Hungary, Italy including Sardinia, Japan, Lesotho, Lithuania, Malta, Nepal, Netherlands, Norway, Oman, Poland, Portugal incl. Azores Islands, Romania, Russia (Yakutia), Serbia, Spain (Canary Islands), South Africa, Sweden, Switzerland, Taiwan, Tunisia, Turkey, and Yemen. First record from Morocco.

##### Habitat.

Collected in a pine forest (*Pinuspinaster*) with herbaceous plants composed of *Trifolium* spp.

#### ﻿ Phytoliriomyza pteridii

Taxon classificationAnimaliaDipteraAgromyzidae

Spencer, 1973

CA05DC74-6602-53A2-A722-96DFE61EE43B

##### Material examined.

Morocco. • 1 ♂; Rif, Fahs-Anjra, Rmel; 27 Feb. 2020; M. Nourti leg.; sweep net; LESCB-R20/36.

##### Distribution.

The species is known from Andorra, Great Britain, Bulgaria, Croatia, France, Germany, Hungary, Ireland, Italy, Montenegro, Poland, Portugal, Scotland, Slovakia, Slovenia, Switzerland, and Yugoslavia. First record from Morocco.

##### Habitat.

It was collected at 372 m in a scrubland with rocky soil of a clay-calcareous habitat crossed by a small stream and bordered by vegetation mainly composed of *Pistacialentiscus*, *Carlinaracemosa*, *Conyzacanadensis*, *Ericaarborea*, and *Cistusmonspeliensis*. On a nearby field, fruit trees such as *Oleaeuropaea* and *Ficuscarica* were growing.

#### ﻿ Phytomyza anemones

Taxon classificationAnimaliaDipteraAgromyzidae

Hering, 1925

4BFFB973-DF4B-5DA6-819F-1EDA3BAC44D2

##### Material examined.

Morocco. • 2 ♂♂, 1 ♀; Rif, Chefchaouen, Bab Tariouant; 31 May 2015; K. Kettani leg.; sweep net; LESCB-R15/07.

##### Distribution.

Denmark, Finland, France, Germany, Great Britain, Greece, Hungary, Ireland, Italy, Lithuania, Malta, Netherlands, Poland, Spain (Mallorca), Sweden, and Yugoslavia. First record from Morocco.

##### Habitat.

Collected in an oak forest at high altitude (*Quercuspyrenaica*).

#### ﻿ Phytomyza clematidis

Taxon classificationAnimaliaDipteraAgromyzidae

Kaltenbach, 1859

412D7AAF-0FDD-518F-ABE9-F5E717AE864B

##### Material examined.

Morocco. • 1 ♂; Rif, Tazia, Dayat Tazia; 12 May 2015; K. Kettani leg.; sweep net; LESCB-R15/05.

##### Distribution.

Recorded from Andorra, Austria, Cyprus, Czech Republic, France incl. Corsica, Germany, Great Britain, Greece, Hungary, Israel, Italy, Lithuania, Maltese Islands, Netherlands, Norway, Portugal, Spain incl. Balearic and Canary Islands, Slovakia, Switzerland, and Turkey. First record from Morocco.

##### Habitat.

Collected on the edge of a pond surrounded by cork oak forest.

#### ﻿ Phytomyza crassiseta

Taxon classificationAnimaliaDipteraAgromyzidae

Zetterstedt, 1860

BEEE3915-AEA9-54D9-A9F8-AE2C92898D37

##### Material examined.

Morocco. • 2 ♂♂; Rif, Bni Darkoul, Oued Souk Lhad; 30 Apr. 2016; F.Z. Bahid leg.; sweep net; LESCB-R16/08. • 6 ♂♂, 4 ♀♀; Rif, Ametrasse, Oued Ametrasse; 30 Apr. 2016; F.Z. Bahid leg.; sweep net; LESCB-R16/09. • 1 ♂, 1 ♀; Rif, Tétouan, Oued Raouz; 18 May 2018; A. Adghir leg.; sweep net; LESCB-R18/26. • 5 ♂♂, 1 ♀♀; Rif, Tazrout, Riba (PNPB); 29 Apr.–22 May 2019; K. Kettani leg.; Malaise trap; LESCB-R19/31. • 4 ♂♂; Rif, Akchour, Pont de Dieu (NPTL); 1 July 2019; M. Nourti leg.; sweep net; LESCB-R19/33. • 1 ♂, 1 ♀; Rif, Oulad Sidi Mansour, Oued Tassikeste; 16 May 2023; K. Kettani leg.; sweep net; LESCB-R23/47.

##### Distribution.

Known from Albania, Andorra, Austria, Belarus, Bulgaria, Croatia, Czech Republic, Denmark, Estonia, Finland, France, Germany, Great Britain, Greece, Hungary, Ireland, Italy, Latvia, Liechtenstein, Lithuania, Montenegro, Netherlands, Norway, Poland, Portugal, Romania, Slovakia, Spain incl. Canary Islands, Sweden, Switzerland. Canada. ON*, QC. USA: CA, ID, IN*, MA, MD, ME, NC, NJ*, NY, PA, VA, WA, WV. Argentina, Chile. Japan, Turkey, and Russia. First record from Morocco.

##### Habitat.

This species has a wide distribution in the Rif across a wide altitudinal range (144–1421 m). It was mainly caught at the banks of rivers bordered by *Neriumoleander*, *Rubusulmifolius*, *Erica* sp., and *Cistus* sp. It was also found in a mixed forest of *Quercusfaginea*, *Quercussuber*, and *Pinuspinaster* at 1421 m altitude.

#### ﻿ Phytomyza notata

Taxon classificationAnimaliaDipteraAgromyzidae

Meigen, 1830

4AF0D5CA-0DA9-544E-8141-6702A4F3CB36

##### Material examined.

Morocco. • 6 ♂♂, 4 ♀♀; Rif, Talassemtane, Maison forestière (NPTL); 7 June–17 July 2014; K. Kettani leg.; Malaise trap; LESCB-R14/04.

##### Distribution.

This species was reported from Belarus, Bulgaria, Czech Republic, Denmark, Finland, France, Germany, Great Britain, Hungary, Italy, Lativia, Lithuania, Poland, Romania, Russia, Serbia, Slovakia, Spain, Sweden, and Switzerland. First record from Morocco.

##### Habitat.

This species was captured in a fir forest (*Abiesmarocana*) at an altitude of 1696 m in Jbel Talassemtane.

#### ﻿ Phytomyza plantaginis

Taxon classificationAnimaliaDipteraAgromyzidae

Robineau-Desvoidy, 1851

B5AE03D4-5E44-55EF-8E5C-A12D42CD9232

##### Material examined.

Morocco. • 1 ♂, 2 ♀♀; Rif, Ametrasse, Oued Ametrasse; 30 Apr. 2016; F.Z. Bahid leg.; sweep net; LESCB-R16/11. • 6 ♂♂, 3 ♀♀; Rif, Talassemtane, Place Espagna (NPTL); 16 Apr. 2018; K. Kettani leg.; sweep net; LESCB-R18/22. • 1 ♂; Rif, Bouhachem, Amsemlil (PNPB); 1 July 2019; K. Kettani leg.; sweep net; LESCB-R19/34. • 2 ♂♂; Atlantic Plain, Sidi Yahya Gharb, forest of Sidi Yahya Gharb; 4 May 2020; M. Doukale leg.; sweep net; LESCB-AP20/35. • 2 ♂♂; Rif, Talembote, Oued Talembote (NPTL); 1 July 2018; K. Kettani leg.; sweep net; LESCB-R18/27. • 1 ♂, 1 ♀; Rif, Tétouan, Khemis Anjra; 27 Mar. 2021; L. Zouhair leg.; sweep net; LESCB-R21/37. • 1 ♂; Rif, Talassemtane, Maison forestière (NPTL); 10 June 2021; K. Menouar & L. Zouhair leg.; sweep net; LESCB-R21/41. • 3 ♂♂; Rif, Afeska, Oued Afeska (NPTL); 10 June 2021; K. Menouar & L. Zouhair leg.; sweep net; LESCB-R21/40.

##### Distribution.

It is recorded from Canada: BC*, ON, QC. USA: Widespread, including HI. Bermuda*. Widespread in Europe, recorded from almost every country including Azores, Canary Islands, Cyprus, Italy, Portugal including Madeira. In the Palaearctic, recorded also from Algeria, Egypt, Iran, Israel, Japan, Kyrgyzstan, Russia, Tunisia, Turkey, and Uzbekistan. It is recorded also from South Africa, Australia, New Zealand, Taiwan, and Thailand. First record from Morocco.

##### Habitat.

This species exhibits a large distribution in the Rif Region of northern Morocco across a wide altitudinal range (11–1683 m) and inhabits a large variety of habitats such as the edge of wetlands (river, peat bog), mixed forest dominated by oak (*Quercussuber*, *Quercuscanariensis*), fir (*Abiesmarocana*), and cedar (*Cedrusatlantica*) at high altitudes and some other vegetation such *Tetraclinisarticulata*, *Eucalyptusglobulus*, *Scolymusmaculatus*, and *Carlinacorymbosa*.

#### ﻿ Phytomyza rufipes

Taxon classificationAnimaliaDipteraAgromyzidae

Meigen, 1830

866EAE96-362A-5A96-A4D7-8E76F33C584C

##### Material examined.

Morocco. • 2 ♂♂, 1 ♀; Rif, Ben Karrich, Oued Mhajrate; 20 July–13 Sep. 2016; K. Kettani leg.; Malaise trap; LESCB-R16/13.

##### Distribution.

It is recorded from Albania, Austria, Belgium, Canada, Croatia (Dalmatia), Czech Republic, Denmark, Egypt, Estonia, Finland, France, Germany, Great Britain, Greece, Iceland, Iraq, Ireland, Italy, Latvia, Lithuania, Netherlands, Norway, Poland, Portugal (Madeira), Russia, Slovakia, Spain (mainland, Canary Islands), Sweden, Switzerland, Turkey, and United Kingdom (Yugoslavia). In the Nearctic Region: Canada (New Brunswick, Newfoundland), USA (California). In the Neotropical Region: Argentina, Colombia. First record from Morocco.

##### Habitat.

Collected on a riverbank bordered by *Armoraciarusticana*, *Alliariapetiolata*, *Brassicaoleracea*, *Diplotaxis* sp., and *Myagrumperfoliatum* and ground vegetation in a pine forest.

#### ﻿ Phytomyza vitalbae

Taxon classificationAnimaliaDipteraAgromyzidae

Kaltenbach, 1872

3D489650-38E3-5022-8676-86C7A3F8FDBA

##### Material examined.

Morocco. • 1 ♂; Rif, Targuist, Bni Bounsar; 1–30 Apr. 2022; K. Kettani leg.; Malaise trap; LESCB-R22/44.

##### Distribution.

The species is very widely distributed, being known from Andorra, Austria, Belgium, Bulgaria, Canada, China, Croatia, Cyprus, Czech Republic, Denmark, Estonia, Finland, France incl. Corsica, Germany, Great Britain, Greece, Hungary, Ireland, Italy, Montenegro, Nepal, Netherlands, New Zealand, Poland, Romania, Russia (Yakutia), Serbia, South Africa, Spain incl. Canary Islands, Sweden, Switzerland. First record from Morocco.

##### Habitat.

Collected in cedar forest (*Cedrusatlantica*).

#### ﻿ Pseudonapomyza confusa

Taxon classificationAnimaliaDipteraAgromyzidae

Zlobin, 1993

F18125FA-7413-5B34-8B82-1B152BA3E1F7

##### Material examined.

Morocco. • 2 ♂♂; Middle Atlas, Ifrane, Zaouiet Ifrane (PNIF); 2 Apr. 2017; Y. Fekrani leg.; sweep net; LESCB-MA17/17. • 1 ♂; Anti-Atlas, Errachidia, Aoufous; 26 Sep. 2022; S. Fekrani leg.; sweep net; LESCB-AA22/43. • 1 ♂; Eastern Morocco, Taourirt, El Aïoun Sidi Mellouk; 9 Jul. 2023; M. Doukale leg.; sweep net; LESCB-EM23/45.

##### Distribution.

It is recorded from Cape Verde Islands, Greece (Crete), Madagascar, and Namibia. First record from Morocco.

##### Habitat.

This species was found at medium altitude in agricultural fields in arid areas in Eastern Morocco as well as in the Anti-Atlas. It was also captured at high altitude in cedar forest (*Cedrusatlantica*) in the Middle Atlas.

#### ﻿ Pseudonapomyza palavae

Taxon classificationAnimaliaDipteraAgromyzidae

Černý, 1998

5A4B6956-80A7-5CC9-8CD1-19614775D827

##### Material examined.

Morocco. • 1 ♂; Eastern Morocco, Taourirt, El Aïoun Sidi Mellouk; 9 July 2023; M. Doukale leg.; sweep net; LESCB-EM24/48.

##### Distribution.

It is recorded from Czech Republic and Hungary. First record from Morocco.

##### Habitat.

This species was found at medium altitude in agricultural fields in arid areas in the Eastern Morocco.

#### ﻿ Pseudonapomyza vota

Taxon classificationAnimaliaDipteraAgromyzidae

Spencer, 1973

266BA3D9-6A44-57E1-BDD6-3EED8FF459D9

##### Material examined.

Morocco. • 1 ♂; Rif, Tazrout, Adrou (PNPB); 14 Jul.–15 Aug. 2013; K. Kettani leg.; Malaise trap; LESCB-R13/03. • 2 ♂♂; Rif, Akchour, Chellal Sghir (NPTL); 7 June–14 July 2017; K. Kettani leg.; Malaise trap; LESCB-R17/18.

##### Distribution.

This species is known from Croatia, Cyprus, France, Greece, Hungary, Italy, Malta, Portugal, Serbia, Spain, Switzerland, and Turkey. First record from Morocco.

##### Habitat.

Collected in an oak-dominated forest and on the banks of a waterfall lined by *Pistacialentiscus*, *Neriumoleander*, *Ficuscarica*, *Mentha* sp., and *Thuya* sp.

## ﻿Discussion

This article presents additions to the Moroccan fauna represented by 30 new records of leaf-miner flies, bringing the total number of Agromyzidae currently known for Morocco to 92 species. This faunal assemblage is subdivided into 11 genera, three genera in Agromyzinae, in which *Agromyza* (*A.anthracina*, *A.conjuncta*, *A.mobilis*, *A.myosotidis*, *A.nigrescens*) dominates in the Rif region and was found in various habitats. The second genus, *Hexomyza* (*H.simplex*) is quite rare, with only one species found in the Atlas region in an agricultural field. The third genus is *Ophiomyia* with the single species *O.pinguis* and 16 individuals present only in the Rif region in various habitats (forest and riverbank). The eight genera in Phytomyzinae are widely distributed across Morocco. *Calycomyza* is newly reported from the country, represented by two species (*C.flavomaculata* and *C.solidaginis*) found in the Rif region on riverbanks at a significant altitude of 1463 m. *Phytomyza* was the prevalent genus in the Rif region and the most diversified with seven species (*Ph.anemones*, *Ph.clematidis*, *Ph.crassiseta*, *Ph.notata*, *Ph.plantaginis*, *Ph.rufipes*, *Ph.vitalbae*). Some other genera were less diversified such as *Cerodontha* (C. (Butomomyza) eucaricis, C. (Dizygomyza) luctuosa, C. (Icteromyza) geniculata), *Liriomyza* (*L.amoena*, *L.brassicae*, *L.strigata*), *Phytoliriomyza* (*P.arctica*, *P.perpusilla*, *P.pteridii*), and *Pseudonapomyza* (*Ps.confusa*, *Ps.palavae*, and *Ps.vota*). The other two genera were less diversified and were found in forest habitats such as *Amauromyza* (A. (Cephalomyza) karli) and *Chromatomyia* (*C.centaurii*). Most obtained species belonged into *Phytomyza* (23,4%) with *Phytomyzacrassiseta* being the most frequent species (38.8%). *Agromyzanigrescens* has a wide distribution in Europe.

These numbers, however, cannot be considered final because further collecting in unexplored areas will undoubtedly bring new additions to the Moroccan fauna

## Supplementary Material

XML Treatment for Agromyza anthracina

XML Treatment for Agromyza conjuncta

XML Treatment for Agromyza mobilis

XML Treatment for Agromyza myosotidis

XML Treatment for Agromyza nigrescens

XML Treatment for Hexomyza simplex

XML Treatment for Ophiomyia pinguis

XML Treatment for Amauromyza (Cephalomyza) karli

XML Treatment for Calycomyza flavomaculata

XML Treatment for Calycomyza solidaginis

XML Treatment for Cerodontha (Butomomyza) eucaricis

XML Treatment for Cerodontha (Dizygomyza) luctuosa

XML Treatment for Cerodontha (Icteromyza) geniculata

XML Treatment for Chromatomyia centaurii

XML Treatment for Liriomyza amoena

XML Treatment for Liriomyza brassicae

XML Treatment for Liriomyza strigata

XML Treatment for Phytoliriomyza arctica

XML Treatment for Phytoliriomyza perpusilla

XML Treatment for Phytoliriomyza pteridii

XML Treatment for Phytomyza anemones

XML Treatment for Phytomyza clematidis

XML Treatment for Phytomyza crassiseta

XML Treatment for Phytomyza notata

XML Treatment for Phytomyza plantaginis

XML Treatment for Phytomyza rufipes

XML Treatment for Phytomyza vitalbae

XML Treatment for Pseudonapomyza confusa

XML Treatment for Pseudonapomyza palavae

XML Treatment for Pseudonapomyza vota

## ﻿Additional information

### Author ORCIDs

Mourad Doukale Daief https://orcid.org/0009-0000-6946-2244

Michael von Tschirnhaus https://orcid.org/0000-0002-1903-4767

Kawtar Kettani https://orcid.org/0000-0003-2361-3996

### Data availability

All of the data that support the findings of this study are available in the main text.
